# Value of a BRAF^V600E^ and lymphocyte subset-based nomogram for discriminating benign lesions from papillary thyroid carcinoma in C-TIRADS 3 and higher nodules

**DOI:** 10.3389/fendo.2025.1608222

**Published:** 2025-08-15

**Authors:** Wenran Zhang, Simei Zeng, Jiaqing Dou, Chenfan Yu

**Affiliations:** Department of Endocrinology, Chaohu Affiliated Hospital of Anhui Medical University, Hefei, China

**Keywords:** PTC, BRAF V600E, NK cell, CD4+ T cell, lymphocyte subpopulation, C-TIRADS, nomogram

## Abstract

**Background:**

The BRAF^V600E^ mutation and lymphocyte subsets may be associated with papillary thyroid carcinoma (PTC). This study established and validated a nomogram model to quantitatively predict the malignant risk of papillary thyroid carcinoma in thyroid nodules classified as C-TIRADS category 3 or higher, providing a reference for precise diagnosis and treatment of these moderately or highly suspicious nodules.

**Methods:**

This retrospective study analyzed 210 patients with thyroid nodules (C-TIRADS ≥3), stratified by fine-needle aspiration biopsy (FNAB) results into benign and PTC groups. Clinical and laboratory parameters were systematically collected for all patients. Variable selection was performed using least absolute shrinkage and selection operator (LASSO) regression, with multicollinearity assessed using variance inflation factors (VIF < 5). Subsequently, significant predictors were incorporated into a multivariate nomogram. Binary logistic regression analysis was employed to identify independent risk factors for PTC following adjustment for potential confounding variables. Internal validation was performed using bootstrap resampling (1,000 iterations) to assess the model’s predictive accuracy, clinical utility, and discriminative ability. Comparative analysis was conducted against the conventional C-TIRADS classification system to evaluate relative performance.

**Results:**

Significant differences were observed between benign thyroid nodules and PTC in age, BRAF^V600E^ genotype, natural killer (NK) cell counts, NK cell percentages, CD4+ T cell percentages, and ultrasound characteristics including size, echogenicity, composition, boundary, and morphology (P < 0.05). Five variables, including age, BRAF^V600E^ genotype, NK cell counts, NK cell%, and CD4+ T cell%, were selected through LASSO regression with collinearity diagnostics for nomogram construction. The model demonstrated excellent discrimination (AUC=0.861, C-index=0.861), good calibration (Hosmer-Lemeshow χ²=6.72, P=0.57), and superior accuracy compared to random prediction (Brier score=0.1061, P<0.05). Decision curve analysis confirmed its clinical utility across relevant probability thresholds. Finally, the comparative analysis demonstrated superior diagnostic performance of the novel nomogram relative to the C-TIRADS system (AUC: 0.862 vs. 0.752; P<0.01).

**Conclusion:**

The BRAF^V600E^-lymphocyte subset nomogram demonstrates robust clinical utility for discriminating benign lesions from PTC in C-TIRADS 3+ thyroid nodules, offering superior diagnostic performance to conventional risk stratification systems.

## 1 Introduction

In recent years, the global incidence of thyroid cancer has escalated rapidly. Over the past three decades, the incidence of thyroid cancer has increased by approximately 300%, with PTC being the primary subtype contributing to this rise (1). This increase is largely attributable to advancements in imaging technologies, which have led to an upsurge in the detection of thyroid nodules. Consequently, there has been a significant rise in the number of patients undergoing nonessential investigations and surgeries. Notably, a substantial proportion of these patients, who are diagnosed with incidentally detected thyroid nodules, ultimately undergo surgery. Yet, the majority of these cases are found to be benign upon final pathology (2).

The BRAF mutation in thyroid tissue is mainly caused by the T→A conversion at 1799 nucleotide of exon 15, that is, the mutation at codon V600E. The BRAF^V600E^ oncoprotein is known to enhance kinase activity, which subsequently activates the MAPK (MEK1/2 and ERK1/2) signaling pathway (3–6). However, the role of the BRAF^V600E^ mutation as an independent predictor of thyroid cancer remains inconclusive in clinical practice.

The overall immune activity in thyroid cancer is elevated, enabling various immune cells to exert both pro- and anti-tumor effects through distinct pathways, thereby shaping a conducive immune landscape. Tumorigenesis recruits peripheral immune cells, such as B cells and T cells, into the tumor microenvironment, facilitating their interaction with tumor cells. Consequently, monitoring changes in circulating immune cells can provide indirect insights into the tumor microenvironment. Given the lack of quantitative indicators for distinguishing benign from malignant thyroid nodules, peripheral blood cytology testing holds significant clinical potential in aiding the diagnosis of papillary thyroid carcinoma.

Ultrasonography serves as the primary modality for thyroid nodule evaluation, providing real-time assessment of morphological features (size, margins, echogenicity) and vascular patterns. When integrated with the C-TIRADS classification system, it enables reliable differentiation of benign and malignant lesions and guides clinical decision-making regarding fine-needle aspiration biopsy or surgical intervention (7). Its non-invasive nature and diagnostic accuracy establish ultrasonography as the first choice for initial screening, diagnostic evaluation, and longitudinal monitoring of thyroid nodules (8).

This study systematically evaluates associations among BRAF^V600E^ mutation status, peripheral blood lymphocyte subsets, and ultrasonographic features with PTC risk in thyroid nodules classified as C-TIRADS categories 3+. With PTC as the primary endpoint, key predictive biomarkers were identified and incorporated into a validated nomogram for clinical risk stratification. We anticipate that these findings will help reduce unnecessary surgeries for benign thyroid nodules while providing a foundation for personalized treatment strategies. Specifically, BRAF^V600E^ mutation testing may guide targeted therapy selection, including BRAF/MEK inhibitors such as dabrafenib/trametinib combination therapy, while lymphocyte subset profiling could provide immunological insights for potential PD-1/PD-L1 checkpoint inhibitor applications in refractory cases.

## 2 Materials and methods

### 2.1 Patient selection

A total of 210 patients with thyroid nodules (C-TIRADS ≥3), diagnosed in Chaohu Hospital affiliated with Anhui Medical University, from September 2023 to May 2025, were retrospectively included in this study. Based on ultrasound-guided FNAB, Bethesda II nodules were classified into the benign nodule group (n=160), while nodules classified as Bethesda V or above were assigned to the PTC group (n=50). *Post hoc* power analysis was performed using R version 4.4.1 (effsize package v0.8.1) and G*Power 3.1.9.7, confirming adequate statistical power (92%) to detect the observed intergroup differences (Cohen’s d = 0.42, α= 0.05). The map of G*Power calculation parameters is provided in **Supplementary Image 1**.

Inclusion Criteria: (a) Thyroid nodules detected by color Doppler ultrasound and classified as TI-RADS category 3 or higher by two experienced sonographers. (b) Availability of detailed and complete clinical data for each patient. (c) Patients whose clinical data were authorized for retrospective analysis by the hospital ethics committee. Exclusion Criteria: (a) Nodules with non-diagnostic FNAB results (Bethesda I), indeterminate cytology (Bethesda III-IV), or cytologically malignant results (Bethesda V-VI) showing non-PTC on final pathology. (b) Patients with comorbidities, such as other infectious or hematologic diseases, that could affect inflammatory markers. (c) Pregnant or lactating women. (d) Patients with a history of other malignant tumors. (e) Patients who have used immunosuppressive drugs within the last six months. (f) Patients who have used anticoagulant drugs in the past week, or those with coagulation abnormalities or hemophilia. The study was reviewed and approved by the Medical Ethics Committee of Chaohu Hospital Affiliated with Anhui Medical University (approval no. KYXM-202409-012).

### 2.2 Patient data and laboratory measurements

Patient demographics were recorded. Peripheral venous blood was collected between 06:00-07:00 the morning post-admission following ≥8h fasting, using standardized tubes by a dedicated team. Medical records and patient interviews confirmed no immune-affecting medications/interventions preceded collection. Analysis of peripheral blood lymphocyte subsets was performed using the BioCyte B5R3 flow cytometer. Fresh anticoagulated whole blood samples were subjected to erythrocyte lysis followed by staining with the following antibody panel: CD3-PE-Cy5/CD4-PE-Cy7/CD8-APC-Cy7/CD16/56-PE for T cell subset analysis and CD19-APC for B cell identification (all antibodies from Zhong Sheng Medical Technology, Hefei, China). Appropriate isotype controls including IgG1-PE (for PE/PE-Cy conjugated antibodies) and IgG2b-APC (for APC/APC-Cy conjugated antibodies) were used to account for nonspecific binding. A minimum of 10,000 lymphocyte events were acquired for each sample. The gating strategy was initiated with the identification of total leukocytes using CD45-FITC in combination with side scatter (SSC) characteristics. Doublets were subsequently excluded based on FSC-A versus FSC-W correlation analysis. Lymphocyte populations were then precisely gated using forward scatter (FSC) and SSC parameters. Data analysis was performed using BioCyteCluster software, with the detailed gating hierarchy illustrated in **Supplementary Image 2**.

### 2.3 BRAF^V600E^ genetic testing protocol

Patients were positioned supine with neck hyperextension for optimal exposure. Following standard disinfection, ultrasound-guided fine-needle aspiration was performed on suspicious thyroid nodules using a multipass technique. The obtained specimens were subjected to DNA extraction using a micro-pathological DNA extraction kit, with DNA quality verified by fluorescence quantification (SLAN-96S PCR system). BRAF^V600E^ mutation status was determined by real-time PCR, with a FAM channel Ct value <38 and ΔCt <9 set as the positive threshold. Samples meeting both criteria were classified as mutation-positive; all others were considered negative.

### 2.4 Ultrasound examination protocol

Standardized thyroid ultrasound examinations were performed using a Samsung HERA W9 color Doppler system with patients in the supine position and neck hyperextended. Systematic evaluation of thyroid nodules was conducted in both transverse and longitudinal planes by at least two board-certified sonographers working independently. Each nodule was carefully assessed for size, echogenicity, composition, multifocality, boundary, and morphology to ensure comprehensive diagnostic evaluation.

### 2.5 Statistical analysis

Statistical power analysis was performed using R 4.4.1 (effsize package v0.8.1) and G*Power 3.1.9.7. All statistical analyses and data visualization were conducted using SPSS 27.0, R 4.4.1, and GraphPad Prism 10.1.2. Normality assessment was performed separately for the benign (n=160) and malignant (n=50) nodule groups. The Kolmogorov-Smirnov test with Q-Q plot visualization was applied to the benign group, while the Shapiro-Wilk test with Q-Q plot analysis was used for the malignant group. Normality was confirmed when Q-Q plot points demonstrated linear alignment with the diagonal reference line. Normally distributed continuous variables were expressed as mean ± standard deviation (χ ± s) and compared using independent samples t-tests. Non-normally distributed data were presented as M (P25, P75) and analyzed using Mann-Whitney U tests. Categorical variables were reported as percentages with between-group comparisons performed using chi-square tests. Binary categorical covariates (including sex, BRAF genotype, and ultrasound features) were dummy-coded (0/1), with complete coding schemes detailed in S3. To identify independent risk factors for PTC, we performed multivariable binary logistic regression analysis with adjustment for potential confounding factors. Before model construction, we rigorously assessed multicollinearity among predictor variables using three complementary diagnostic measures: (1) Pearson correlation coefficients (|r| >0.7 indicating potential collinearity), (2) tolerance values (<0.1 suggesting collinearity), (3) variance inflation factors (VIF >5 indicating collinearity).

The initial set of independent variables included demographic characteristics (age and sex), BRAF^V600E^ mutation status, clinical laboratory parameters (lymphocyte subsets including T cell percentages, absolute T cell counts, CD4+ T cell counts, CD8+ T cell counts, NK cell counts, B cell counts, CD4+ T cell percentages, CD8+ T cell percentages, NK cell percentages, B cell percentages, and CD4+/CD8+ ratio), and comprehensive ultrasound characteristics (including nodule size, echogenicity, composition, multifocality, boundary, and morphology). Variable selection was performed using LASSO regression in R 4.4.1 (glmnet v4.1–8 package), with the optimal lambda value selected through 10-fold cross-validation. Significant predictors (β ≠ 0) were incorporated into a nomogram (rms v7.0–0 and Hmisc v5.2–2 packages) to estimate PTC probability. Internal validation included bootstrap resampling (1000 iterations) to assess discrimination (ROC analysis), calibration (Hosmer-Lemeshow test, calibration curves), model fit (Brier score), and clinical utility (decision curve analysis) using relevant R packages. The software packages used in these analyses include “car”(v3.1-3), “survival”(v3.8-3), “pROC”(v1.18.5), “tcltk”(v4.4.1), “ResourceSelection”(v0.3-6), “DescTools”(v0.99.60), “rmda”(v1.6), “Rcpp”(v1.0.14). Finally, the performance of the prediction model was compared with the TI-RADS classification system by receiver operating characteristic (ROC) curve analysis using GraphPad Prism 10.1.2, with the area under the curve (AUC) serving as the primary comparative metric.

## 3 Results

### 3.1 Comparative analysis of clinical characteristics between PTC and benign thyroid nodules

Patients with PTC demonstrated significantly younger age, lower NK cell counts and percentages, and higher CD4+ T cell percentages compared to those with benign nodules. The PTC group exhibited a higher prevalence of BRAF^V600E^ mutations and ultrasonic features, including smaller size (<10mm), hypoechogenicity, solid composition, irregular margins, and irregular morphology. No statistically significant differences were observed in gender distribution, T cell absolute counts, T cell percentages, CD4+ T cell counts, CD8+ T cell counts, B cell counts, CD8+T cell percentages, B cell percentages, and CD4+/CD8+ or multifocality between the groups (**Table 1**).

**Table 1:** Comparative analysis of clinical characteristics between the two groups.

Characteristics	Benign nodule group	PTC group	Z/t/x^2^	P
Age	55.50 (50.00,60.00)	52.00 (41.75,58.00)	-2.187	0.029
Sex			0.371	0.542
Woman	131 (81.9)	39 (78.00)		
man	29 (18.1)	11 (22.00)		
BRAF^V600E^			71.377	<0.001
wild type	150 (93.80)	20 (40.00)		
mutant type	10 (6.30)	30 (60.00)		
T cell %	70.69±9.01	72.31±8.04	-1.142	0.255
T cell absolute counts	1100.28 (823.25,1332.00)	1034.50 (809.00,1267.91)	-0.809	0.418
CD4+ T cell counts	628.50 (482.75,802.46)	595.00 (512.25,809.88)	-0.109	0.913
CD8+T cell counts	335.86 (247.25,478.75)	328.95 (247.00,405.75)	-0.697	0.486
NK cell counts	212.50 (131.25,376.00)	167.50 (119.75,248.25)	-2.193	0.028
B cell counts	173.50 (120.50,246.25)	181.09 (145.25,251.00)	-0.823	0.411
CD4+T cell %	42.04±8.00	44.61±6.03	-2.094	0.037
CD8+T cell %	23.26 (17.79,28.84)	23.20 (20.09,26.60)	-0.229	0.819
NK cell %	14.61 (9.27,22.13)	11.52 (8.04,16.60)	-2.293	0.022
B cell %	11.74 (8.72,14.95)	12.93 (10.80,16.18)	-1.776	0.076
CD4+/CD8+	1.78 (1.35,2.46)	1.89 (1.48,2.34)	-0.753	0.451
Ultrasound features				
size			5.966	0.015
<10mm	36 (22.5)	20 (40.0)		
≥10mm	124 (77.5)	30 (60.0)		
echogenicity			4.432	0.035
Non-hypoechoic	50 (31.3)	8 (16.0)		
hypoechoic	110 (68.8)	42 (84.0)		
composition			5.091	0.024
Non-solid	40 (25.0)	5 (10.0)		
solid	120 (75.0)	45 (90.0)		
multifocality			0.025	0.874
Solitary	21 (13.1)	7 (14.0)		
Multiple	139 (86.9)	43 (86.0)		
boundary			5.446	0.020
clear	138 (86.3)	36 (72.0)		
obscure	22 (13.8)	14 (28.0)		
Morphology			5.612	0.018
regular	131 (81.9)	33 (66.0)		
irregular	29 (18.1)	17 (34.0)		

T cell %, T cell percentages; CD4+ T cell %, CD4+ T cell percentages; CD8+ T cell %, CD8+ T cell percentages; NK cell %, NK cell percentages; B cell %, B cell percentages; CD4+/CD8+, CD4+T cell counts/CD8+T cell counts ratio; Non-hypoechoic, isoechoic or hyperechoic; Non-solid, cystic or spongiform or mixed.

### 3.2 Feature selection using LASSO regression

**Figure 1:**
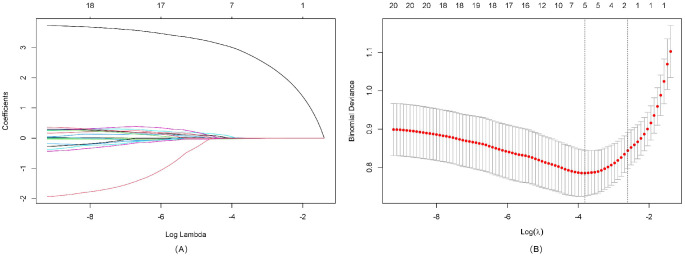
LASSO regression analysis for feature selection. **(A)** LASSO coefficient path diagram: The x-axis represents log-lambda, reflecting the degree of regularization. It shows that as the lambda value increases, the coefficient values shrink gradually to zero, and the number of retained variables decreases. **(B)** LASSO regularization path diagram: The x-axis is log(lambda); the upper axis denotes the number of non-zero coefficients, while the left y-axis represents the binomial deviance. This graph demonstrates the variation in binomial deviance with different lambda values. The two vertical lines represent the optimal lambda values selected by cross-validation. The left dashed line is lambda.min, where lambda minimizes the binomial deviance, and the right dashed line is lambda.1se, where lambda corresponds to the most regularized model within one standard error of the minimum deviance.

We performed LASSO regression analysis incorporating demographic characteristics (age, gender), sonographic features (nodule size, echogenicity, composition, multifocality, boundary, and morphology), BRAF^V600E^ mutation status, and comprehensive lymphocyte subset profiles (including T cell percentages, T cell absolute counts, CD4+ T cell counts, CD4+ T cell percentages, CD8+ T cell counts, CD8+ T cell percentages, NK cell counts, NK cell percentages, B cell counts, B cell percentages and CD4+/CD8+ radio) to identify the most discriminative predictors of thyroid nodule malignancy. Through 10-fold cross-validation, the optimal penalty parameter (lambda = lambda.min) yielded a parsimonious model containing five key variables: patient age, BRAF^V600E^ mutation status, NK cell counts, NK cell percentages, and CD4+ T cell percentages. The variable selection process is visualized in **Figure 1A** (coefficient profiles), while **Figure 1B** displays the cross-validation curve demonstrating model optimization. The assignment table for categorical variables is provided in **Supplementary Table 1**.

### 3.3 Collinearity assessment

Following LASSO regression selection, we evaluated potential multicollinearity among the identified predictors (age, BRAF^V600E^ genotype, NK cell counts, NK cell percentages, and CD4+ T cell percentages) using Pearson correlation analysis and VIF diagnostics. Initial correlation analysis revealed a possible association between NK cell counts and NK cell percentages (r=0.763, P<0.01), exceeding our predefined threshold of |r|>0.7 for potential collinearity (**Table 2**). Subsequent multicollinearity testing demonstrated acceptable tolerance values (>0.1) and VIF (<5) for all variables (**Table 3**), confirming the absence of significant multicollinearity in our final model.

**Table 2:** Correlation analysis of predictor variables.

Characteristics	Age	BRAF^V600E^ genotype	NK cell counts	NK cell %	CD4+T cell %
Age	1.000				
BRAF^V600E^ genotype	-0.069	1.000			
NK cell counts	0.128	0.008	1.000		
NK cell %	0.157	-0.002	0.763*	1.000	
CD4+T cell %	0.006	0.044	-0.378	-0.483	1.000

NK cell %, NK cell percentages; CD4+ T cell %, CD4+ T cell percentages; *represents P<0.01.

**Table 3:** Collinearity diagnosis.

Characteristics	Tolerance	VIF
Age	0.961	1.041
BRAF^V600E^ genotype	0.992	1.008
NK cell counts	0.417	2.395
NK cell %	0.369	2.714
CD4+T cell %	0.757	1.321

NK cell %, NK cell percentages; CD4+ T cell %, CD4+ T cell percentages; VIF, variance inflation factors.

### 3.4 Multivariate regression analysis of thyroid nodules with C-TIRADS category 3 and above

Multivariate logistic regression analysis (forward conditional method) was performed with five variables: age, BRAF^V600E^ genotype, NK cell counts, NK cell percentages, and CD4+ T cell percentages. The analysis identified BRAF^V600E^ genotype as an independent risk factor for PTC (OR = 36.088, 95% CI [12.984~100.297], p < 0.001), indicating a significantly elevated malignancy risk in mutation-positive patients. Conversely, age (OR = 0.962, 95% CI [0.926~0.999], p = 0.045) and NK cell counts (OR = 0.995, 95% CI [0.992~0.998], p = 0.002) emerged as protective factors. Neither NK cell percentage nor CD4+ T cell percentage retained statistical significance in the final model. Model fit was confirmed by Hosmer-Lemeshow testing (χ² = 7.198, p = 0.515), with an overall classification accuracy of 87.6% (**Table 4**).

**Table 4:** Multivariate regression analysis of thyroid nodules with C-TIRADS category 3 and above.

Characteristics	B	S.E	Wald	df	P	OR (95% CI)
Age	-0.039	0.019	4.032	1	0.045	0.962 (0.926~0.999)
BRAF^V600E^ genotype	3.586	0.522	47.276	1	0.000	36.088 (12.984~100.297)
NK cell counts	-0.005	0.002	9.365	1	0.002	0.995 (0.992~0.998)
Constant	1.038	1.035	1.006	1	0.316	2.823

### 3.5 Construction and validation of the nomogram model

#### 3.5.1 Construction of the nomogram

**Figure 2:**
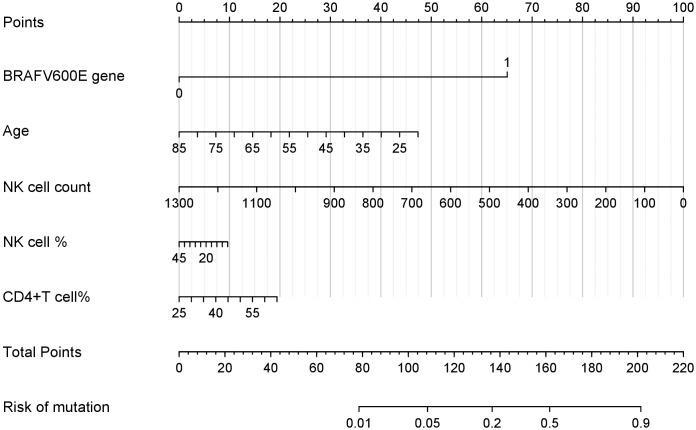
Nomogram for the risk of PTC in thyroid nodules with C-TIRADS category 3 or above. This nomogram integrates multiple predictors identified by lasso to estimate the probability of PTC risk in thyroid nodules classified as C-TIRADS category 3 or above. Each variable is assigned a point value based on its contribution to the model. The total points (sum of individual variable points) correspond to the predicted mutation risk at the bottom scale.

Using the independent predictors identified by LASSO regression (age, BRAF^V600E^ genotype, NK cell counts, NK cell percentages, and CD4+ T cell percentages), we constructed a clinically applicable nomogram (**Figure 2**) to estimate the individualized risk of malignancy in thyroid nodules classified as C-TIRADS category 3 or above.

#### 3.5.2 Curve of calibration

**Figure 3:**
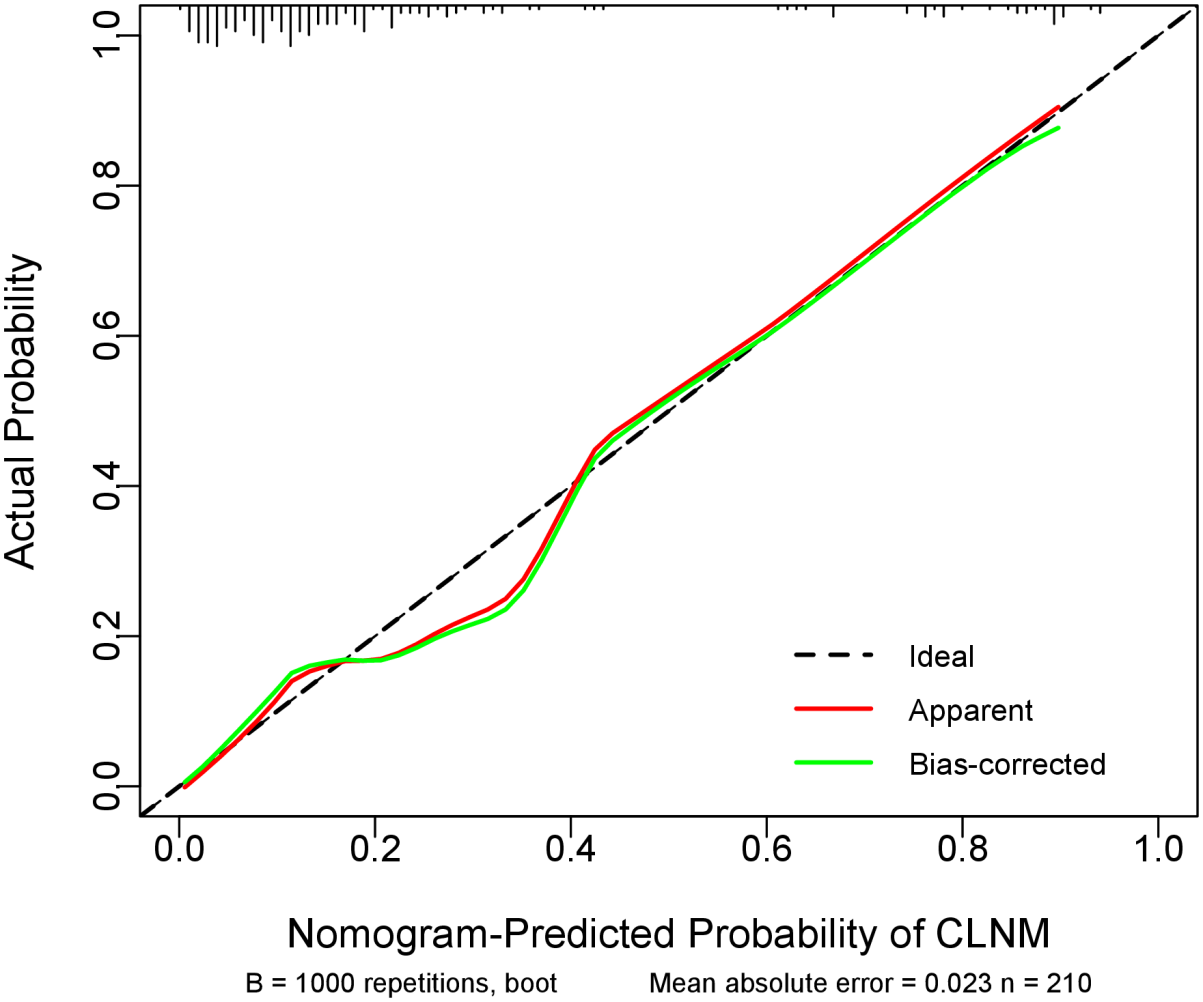
Calibration curve of the prediction model. The diagonal dotted line representsthe ideal prediction by the perfect nomogram. The green solid ine represents the performance of the nomogram, The closer the green solid line is to the diagonal dotted line, the stronger the predictive ability of the model. The red solid ine indicates the apparent predictive accuracy.

Internal validation using 1000 bootstrap resamples demonstrated excellent model discrimination, with an AUC and concordance index (C-index) of 0.861 (95% CI: 0.859–0.863). The Hosmer-Lemeshow goodness-of-fit test (χ² = 6.72, p = 0.57) and calibration curve analysis (MAE=0.023) (**Figure 3**) indicated strong agreement between predicted and observed outcomes, confirming robust model calibration.

#### 3.5.3 Brier score

**Figure 4:**
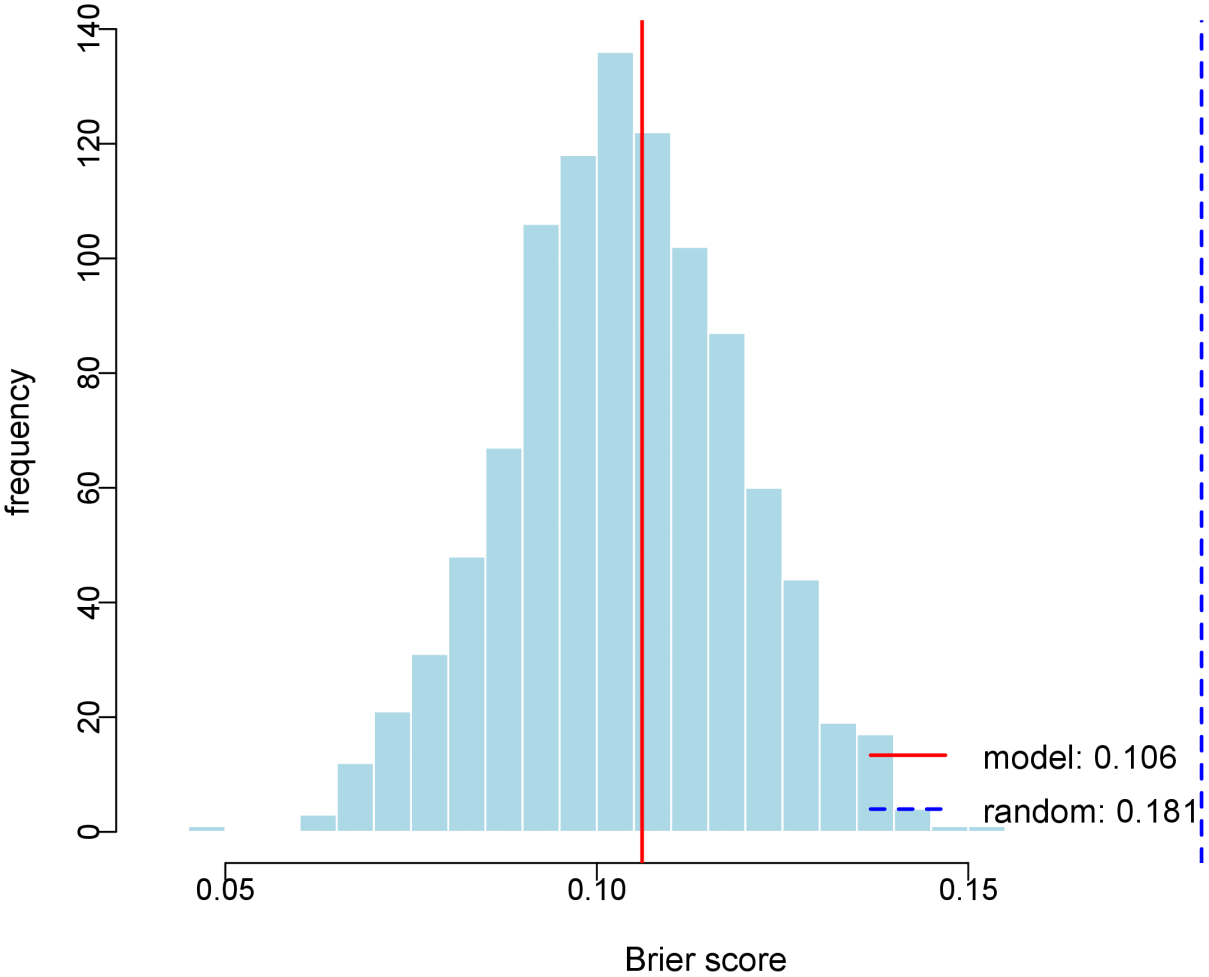
Histogram of Bootstrap Brier Score Distribution. The figure depicts the distribution of Brier scores derived from 1000 bootstrap replications. The red solid line represents the Brier score of the predictive model (0.106), and the blue dashed line indicates the Brier score of the random model (0.181).

Internal validation via 1000 bootstrap resamples demonstrated superior predictive accuracy of our model (Brier score = 0.1061) compared to random guessing (Brier score = 0.1814, 95% CI: 0.073-0.134, **Figure 4**). These results confirm the model’s robust discriminative ability for distinguishing benign lesions from PTC in thyroid nodules classified as C-TIRADS category 3 or above.

#### 3.5.4 Decision curve analysis

**Figure 5:**
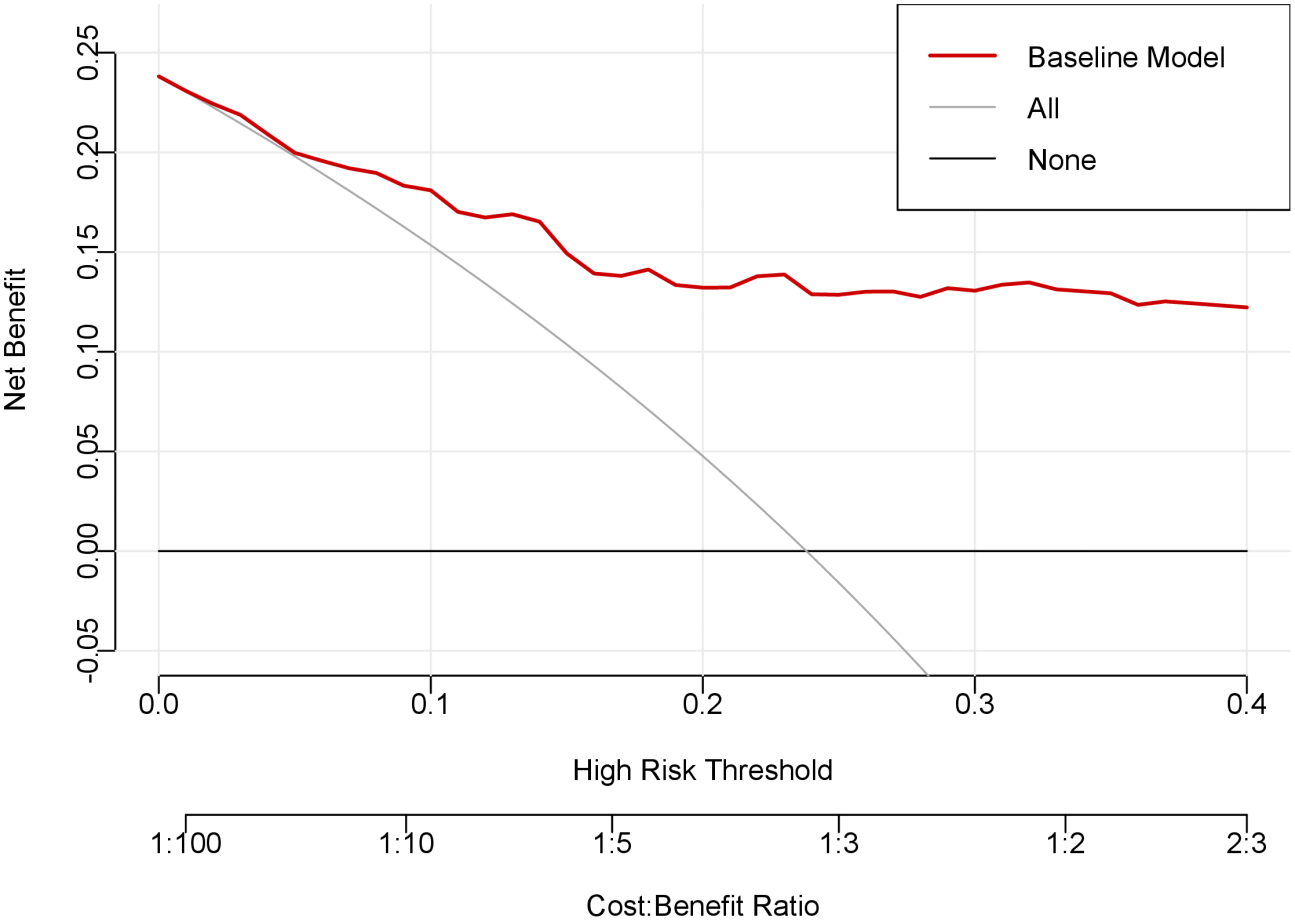
Decision curve analysis of the prediction model. The x-axis of this curve represents the risk threshold probability, while the y-axis indicates the net clinical benefit derived from decision-making based on the prediction model. The red curve denotes the net benefit achieved by applying the nomogram, the gray curve illustrates the net benefit of the “treat-all” strategy, and the black curve shows the net benefit of the “treat-none” strategy.

Decision curve analysis demonstrated the superior clinical utility of our prediction model across a wide range of risk thresholds (**Figure 5**). The model’s net benefit curve substantially exceeded both the “treat-all” and “treat-none” reference strategies, indicating robust clinical applicability for decision-making in thyroid nodule management.

### 3.6 Comparative performance of prediction model versus C-TIRADS system

**Figure 6:**
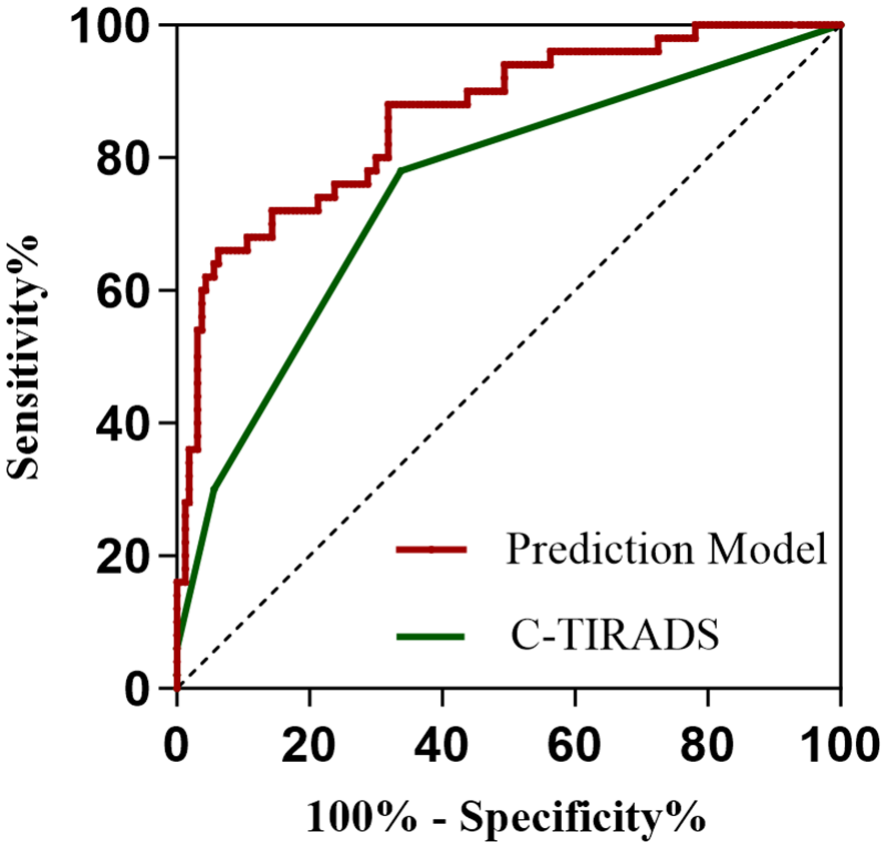
ROC curves of the prediction model and the C-TIRADS system. The area under the curve (AUC) serves as an indicator of predictive accuracy and generalization capability.

All 210 thyroid nodules (160 benign, 50 PTC) were classified according to C-TIRADS guidelines, demonstrating progressively increasing malignancy rates across categories: 9.40% (11/117) for category 3, 34.78% (24/69) for 4a, 57.14% (12/21) for 4b, 100% (2/2) for 4c, and 100% (1/1) for category 5 (P<0.001, **Table 5**). For quantitative analysis, the C-TIRADS was assigned numerical codes as follows: C-TIRADS 3 = 0, C-TIRADS 4a = 1, C-TIRADS 4b = 2, C-TIRADS 4c = 3, and C-TIRADS 5 = 4. ROC curve analysis was performed to compare the diagnostic efficacy between our prediction model and the C-TIRADS for differentiating benign and malignant thyroid nodules. The developed prediction model for assessing PTC risk in C-TIRADS category 3+ demonstrated significantly superior discriminative ability, with an AUC of 0.862 (95% CI: 0.802-0.922; P<0.001). At the optimal cutoff value of 0.320, the model achieved a specificity of 93.7% and sensitivity of 66.0%, yielding a Youden’s index of 59.7%. In comparison, the C-TIRADS showed an AUC of 0.752 (95% CI: 0.672-0.832; P<0.001), with 0.5 (corresponding to category ≥4a) as the optimal cutoff (specificity: 66.25%; sensitivity: 78.0%; Youden’s index: 44.25%). These results demonstrate that our novel prediction model significantly outperforms the conventional C-TIRADS system in discriminating benign lesions from PTC among C-TIRADS category 3 and above nodules, suggesting its potential clinical utility for improved decision-making in thyroid nodule management (**Figure 6**).

**Table 5:** C-TIRADS classification of thyroid nodules.

C-TIRADS Classification	Nodule numbers	Benign nodules	Malignant nodules
C-TIRADS 3	117	106 (90.60)	11 (9.40)
C-TIRADS 4a	69	45 (65.21)	24 (34.78)
C-TIRADS 4b	21	9 (42.86)	12 (57.14)
C-TIRADS 4c	2	0 (0.00)	2 (100)
C-TIRADS 5	1	0 (0.00)	1 (100)
x^2^	38.596		
P	<0.001		

## 4 Discussion

Building upon prior research, this study innovatively investigated the diagnostic value of lymphocyte subsets in differentiating benign from malignant thyroid nodules. Our analysis revealed that NK cell counts serve as a protective factor against malignancy in thyroid nodules classified as C-TIRADS category 3 or higher. We developed a novel nomogram model incorporating five key variables (BRAF^V600E^ genotype, NK cell counts, NK cell percentage, CD4+ T cell percentage, and age) to predict malignancy risk in these nodules. Validation demonstrated that the model exhibits excellent discriminative ability, strong calibration, and superior predictive performance compared to the traditional C-TIRADS. These findings suggest significant potential for clinical translation in thyroid nodule risk stratification.

The Bethesda System for Reporting Thyroid Cytopathology (TBSRTC) is a standardized classification system for thyroid FNA cytology, demonstrating high sensitivity and specificity in diagnosing thyroid cancer (9). Previous studies have confirmed the diagnostic accuracy of TBSRTC categories II to VI (10). Accordingly, this study enrolled 210 patients with thyroid nodules classified as C-TIRADS category 3 or above. Based on FNAB results, nodules categorized as Bethesda II were assigned to the benign group, while those classified as Bethesda V or higher and subsequently confirmed as PTC through postoperative histopathological examination comprised the PTC group. The study objectives were to identify potential risk factors associated with PTC development and establish a risk prediction model for discriminating between benign thyroid nodules and PTC, thereby facilitating more accurate clinical diagnosis and treatment decision-making.

This study demonstrated that the BRAF^V600E^ mutation serves as an independent risk factor for PTC. As a major oncogenic driver, the BRAF^V600E^ mutation is present in approximately 60% of PTC cases (11). Located on chromosome 7q34, the BRAF gene encodes a threonine/serine protein kinase belonging to the RAF family. As a critical component of the MAPK signaling pathway, BRAF regulates cell growth, proliferation, and apoptosis. Dysregulation of this pathway due to BRAF mutations can contribute to tumorigenesis (12).

Our analysis identified age as an independent protective factor against PTC, corroborating existing epidemiological evidence (13). The observed inverse association may be attributed to age-related biological mechanisms including telomere attrition, stem cell depletion, impaired macroautophagy, and cellular senescence - processes known to mediate environment-dependent tumor suppression and potentially attenuate carcinogenesis risk (14, 15).

The tumor microenvironment (TME) comprises the cellular and molecular milieu supporting tumorigenesis and progression. This complex ecosystem includes not only tumor cells themselves, It also includes innate immune cells (such as macrophages, mast cells, granulocytes, myeloid-derived suppressor cells, dendritic cells, natural killer cells, etc.), adaptive immune cells (such as T lymphocytes, B lymphocytes), interstitial fibroblasts, extracellular matrix, vascular and lymphatic networks, and inflammatory factors and growth produced by them and tumor cells through autocrine or paracrine Factors, chemokines and other non-cellular components. These components engage in dynamic crosstalk that collectively governs tumor progression and metastatic dissemination.

Specific cellular or molecular features in TME have been shown to be independent prognostic factors. Accumulating evidence indicates that lymphocytic infiltration is associated with favorable outcomes across various cancer types (16–19). During thyroid cancer progression, patients exhibit systemic immune activation accompanied by a marked expansion of pro-tumorigenic immune cell populations (20). Notably, PTC with robust pericarcinoma lymphocytic infiltration exhibits an improved prognosis compared to PTC lacking such infiltration (21). This phenomenon may arise from lymphocyte-derived cytokine-mediated tumor suppression or cancer cell evasion of immune surveillance via mechanisms such as major histocompatibility complex (MHC) modulation. Peripheral blood immune cells serve as both systemic immune indicators and active participants in tumor immunomodulation. These cells migrate to tumor sites where they influence local immune responses through multiple mechanisms including chemotaxis, cell-cell signaling, and systemic immune modulation, ultimately affecting tumor progression and therapeutic outcomes (22). Quantitative monitoring of circulating lymphocyte populations may offer valuable insights into cancer development and prognostic evaluation. Given these observations, we hypothesized that peripheral blood lymphocyte subset alterations might influence PTC development. NK cells, critical effectors of innate immunity, contribute substantially to antitumor responses and cancer immunosurveillance (23, 24). Depletion of NK cells has been associated with elevated tumor susceptibility (25, 26). Our findings demonstrate that NK cells serve as protective factors in PTC. Specifically, a 1-cell/μL increment in NK cell counts was associated with a 0.5% reduction in PTC risk. This finding is consistent with previous reports in lung adenocarcinoma (27), colorectal cancer (28), gastric cancer (29), and melanoma (30). Higher tumor-infiltrating NK cell abundance was significantly associated with better clinical outcomes in different types of malignancies. This may be related to the fact that NK cells achieve tumor control by recognizing and killing tumor cells and promoting adaptive T-cell immune responses (31–33).

Our analysis of thyroid nodules classified as C-TIRADS category 3 or higher revealed a distinct pattern in CD4+ T-cell distribution. While absolute CD4+ T-cell counts showed no significant difference between benign lesions and PTC, the relative percentages of CD4+ T cells were markedly elevated in PTC patients compared to their benign counterparts. Notably, this parameter persisted as an independent predictive factor in our LASSO-optimized risk stratification model. These findings contrast with previous reports (34), suggesting complicated thyroid-specific immunomodulatory mechanisms. An investigation in oligometastatic non-small cell lung cancer (NSCLC) has demonstrated significantly elevated peripheral CD4+ T-cell levels in patients with brain metastases compared to healthy controls (35). These findings suggest CD4+ T cells may contribute to anti-tumor immune responses, potentially through specific mechanisms. Substantial interindividual variability exists in CD4+ T cell measurements, with absolute counts being particularly susceptible to confounding factors including active infections, pharmacologic interventions, chronic inflammatory conditions, leukocytosis, and post-splenectomy status (36). Notably, while these factors may significantly elevate absolute CD4+ counts, they generally induce only marginal fluctuations in CD4+ percentages. Consequently, the CD4+ percentages appear to represent a more stable immunological indicator.

Mantovani et al. demonstrated that both innate and acquired immune responses can either promote cancer initiation and tumor progression or exert anticancer effects (37). However, current evidence on the diagnostic value of peripheral blood lymphocyte subsets in PTC remains limited and inconsistent. Some studies report no significant differences in the percentages of CD8+ T cells, CD4+ T cells, and NK cells in peripheral blood between PTC patients and those with benign thyroid nodules (38). In contrast, other research has identified a higher proportion of T cells, CD3+ T cells, CD4+ T cells, CD8+ T cells, and regulatory T cells (Tregs) in the peripheral blood of PTC patients compared to benign cases (34), correlating with PTC aggressiveness (39). Investigations focusing on lymphocyte subset infiltration within thyroid tissue have consistently linked CD8+ T cells, CD4+ T cells, and B cells to PTC (38, 40–45). Notably, our findings align with prior reports (45) indicating the minimal influence of peripheral blood T cells and B cells on PTC development. The observed discrepancies may reflect methodological variations across studies, particularly regarding BRAF^V600E^ mutation status adjustment, which was adequately controlled in our analysis. Moreover, we employed high-resolution flow cytometry to precisely quantify both absolute lymphocyte counts and subset percentages simultaneously, a methodological advancement over conventional morphological analysis that significantly improves measurement accuracy. The compartmentalized distribution of lymphocyte subsets - with distinct peripheral blood profiles contrasting sharply with tissue infiltration patterns - suggests a model where T and B lymphocytes undergo primary activation within the thyroid tumor microenvironment before systemic dissemination, potentially accompanied by either clonal expansion or functional exhaustion. Consequently, while intratumoral T cells exhibit pronounced activation in PTC, peripheral blood alterations may remain subclinical (38, 46).

In this study, we employed LASSO regression to identify significant predictors for PTC risk among thyroid nodules classified as C-TIRADS category 3 or above. To facilitate clinical implementation, we constructed a nomogram that visually represents the model parameters, enabling intuitive and individualized risk stratification for PTC. The nomogram demonstrated favorable discrimination, accuracy, and clinical utility. Comparative analysis with the conventional C-TIRADS system revealed superior performance, offering enhanced clinical value for individualized risk assessment of thyroid nodules.

This study has several limitations that warrant consideration. Firstly, this study employed peripheral blood analysis to characterize lymphocyte subsets while recognizing that circulating immune profiles may not accurately represent the local thyroid tissue microenvironment, particularly regarding tumor-infiltrating lymphocytes (TILs) and tissue-resident NK cells. The biological interpretation of our findings requires consideration of potential discrepancies in immune cell migration and phenotype between peripheral circulation and thyroid tissue. Furthermore, peripheral lymphocyte proportions may be influenced by systemic inflammatory status and hormonal fluctuations. Future investigations should incorporate single-cell RNA sequencing of FNA specimens or multiplex immunofluorescence analysis to directly evaluate spatial lymphocyte distribution and functional states within thyroid nodules, thereby validating the tissue-level applicability of our model.

Secondly, the relatively small sample size from a single institution restricted the more comprehensive variable analysis to prevent overfitting, and although LASSO regression was used for variable selection, potential residual confounders may remain. Importantly, *post-hoc* power analysis confirmed our study maintained 92% statistical power to detect moderate effect sizes, ensuring robust reliability of the reported associations. Additionally, the scarcity of Bethesda VI nodules necessitated combining categories V and VI for statistically reliable analysis. These constraints highlight the need for future multi-center studies with larger cohorts to enable stratified analyses and external validation. In subsequent research, we plan to collect comprehensive clinical data (including peripheral blood lymphocyte subsets, C-TIRADS classification, BRAFV600E genotype, and histopathological type) from a minimum of 500 thyroid nodule patients across two provincial tertiary hospitals over a 24-month period. This multicenter validation study will assess the model’s generalizability across diverse populations and the consistency of the NK cell counts-PTC association in different demographic and clinical subgroups.

Thirdly, the cross-sectional nature of our study design precludes causal inference regarding the relationship between identified factors and PTC development. Longitudinal studies would be required to establish temporal relationships and causality.

Finally, while FNAB represents our primary diagnostic modality, its accuracy is inherently influenced by several technical and biological factors including nodule characteristics (size, location), sampling technique, and cytological preparation quality. To maximize diagnostic reliability, we implemented rigorous quality control measures: all procedures were performed by experienced senior clinicians (chief or deputy chief physicians), with strict adherence to the Bethesda System for Reporting Thyroid Cytopathology. This standardized approach helped mitigate potential variability, though the intrinsic limitations of cytological interpretation remain an acknowledged constraint of this study.

## 5 Conclusion

This study developed and validated a clinically useful nomogram incorporating BRAF^V600E^ genotype and lymphocyte subsets, which demonstrated superior diagnostic accuracy in differentiating benign lesions from PTC among C-TIRADS category 3 and higher nodules. The model may help refine risk stratification and reduce unnecessary invasive interventions in indeterminate cases.

## Data Availability

The raw data supporting the conclusions of this article will be made available by the authors, without undue reservation.

## Supplementary material

Supplementary Image 1 The map of G*Power calculation parameters.

Supplementary Image 2 Representative flow cytometry dot plots. (A) represents the distribution of all cells, (B) represents the distribution of CD45+ cells, (C) represents the distribution of CD3+ T cells, (D) represents the distribution of CD4+ cells, (E) represents the distribution of CD8+ T cells, (F) represents the distribution of B (CD19+) cells, and (G) represents the distribution of NK cells.

Supplementary Table 1 Assignment Table of variables.
