# Supplementary figures and images for: Value of a BRAFV600E and lymphocyte subset-based nomogram for discriminating benign lesions from papillary thyroid carcinoma in C-TIRADS 3 and higher nodules
Source: Front Endocrinol (Lausanne). 2025 Aug 15;16:1608222. doi: 10.3389/fendo.2025.1608222 (PMC12394038; doi:10.3389/fendo.2025.1608222)

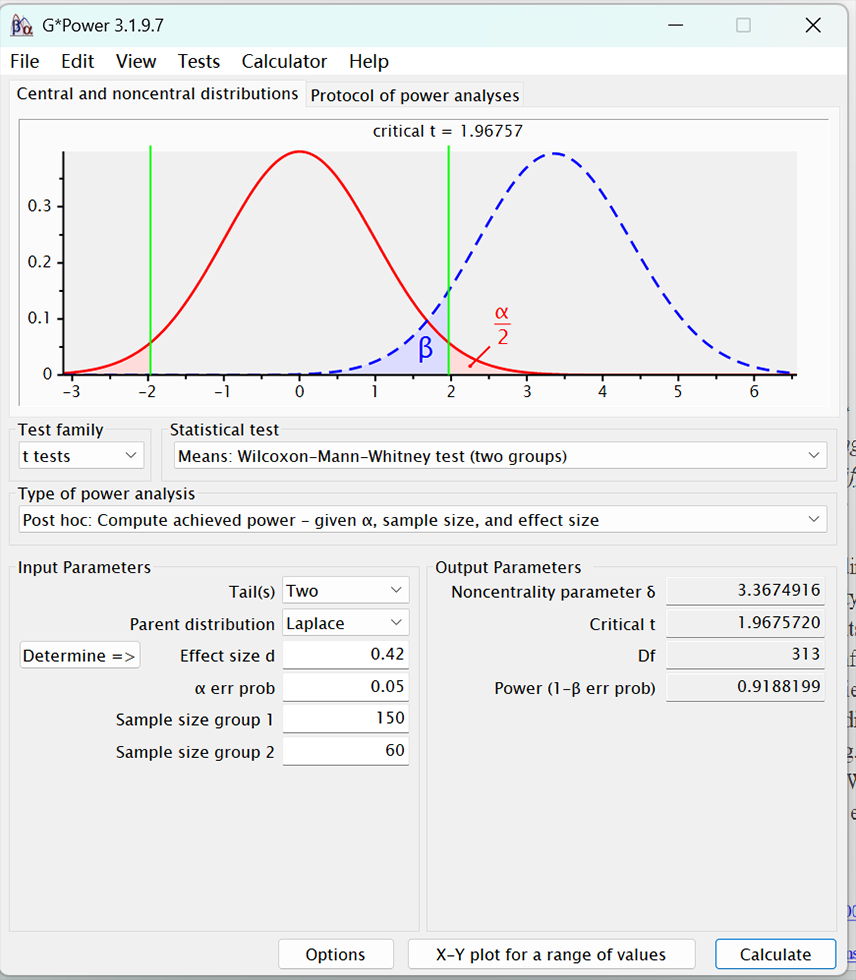

Supplement: Supplementary Image 1 — The map of G*Power calculation parameters. [file Image1.tiff]

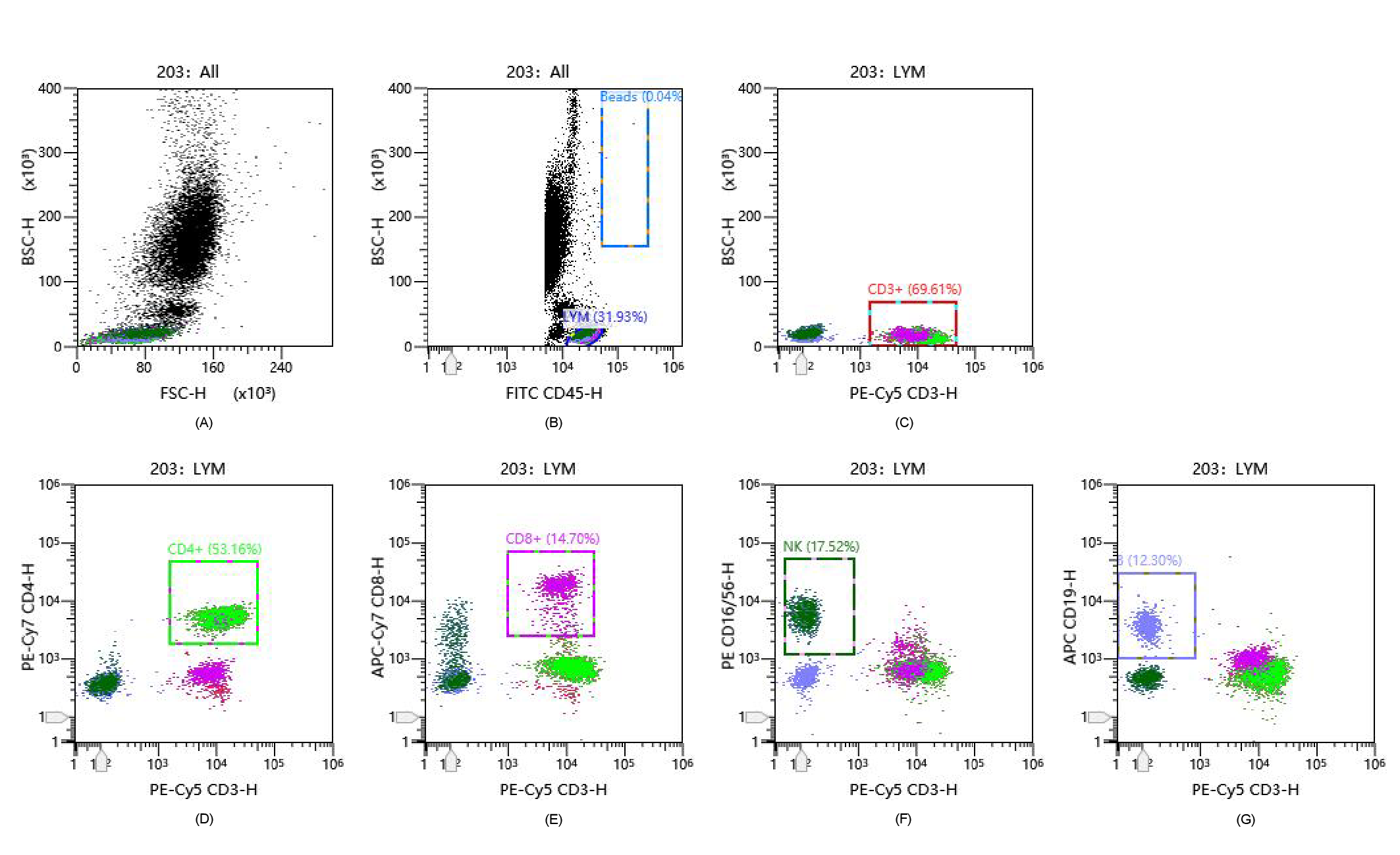

Supplement: Supplementary Image 2 — Representative flow cytometry dot plots. (A) represents the distribution of all cells, (B) represents the distribution of CD45+ cells, (C) represents the distribution of CD3+ T cells, (D) represents the distribution of CD4+ cells, (E) represents the distribution of CD8+ T cells, (F) represents the distribution of B (CD19+) cells, and (G) represents the distribution of NK cells. [file Image2.tiff]

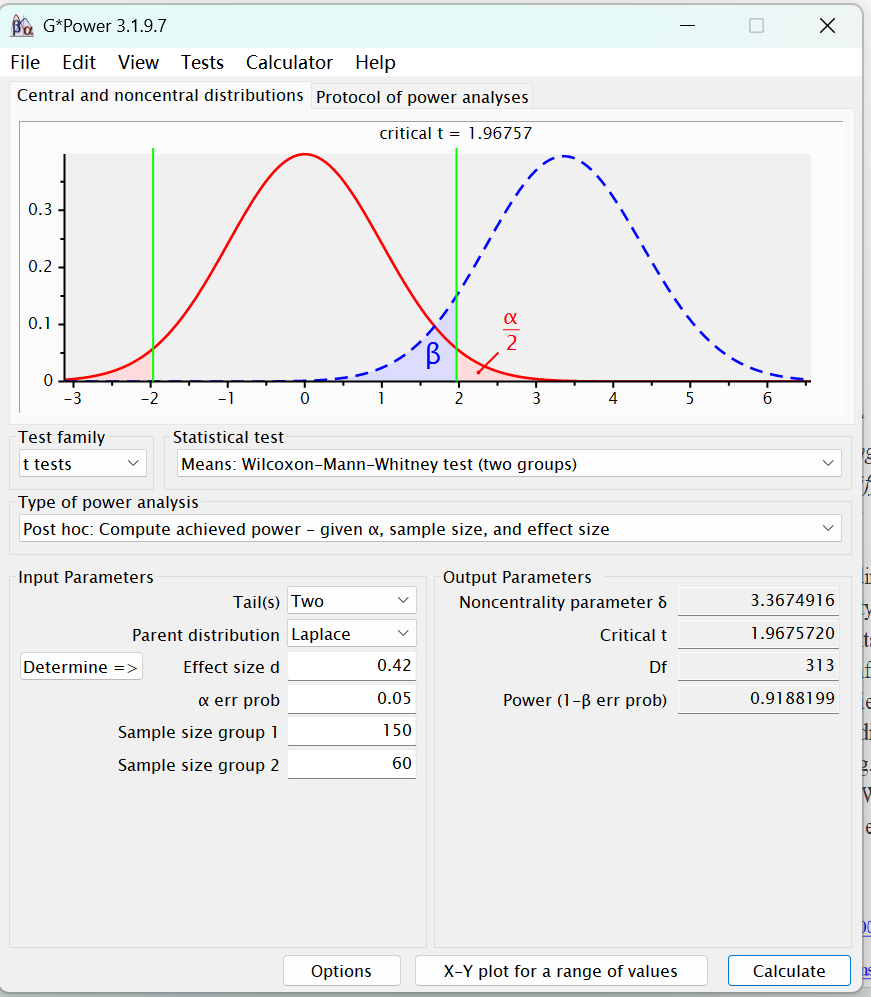

Supplement: Supplementary file 4 [file DataSheet1.zip › S1-3/S2.png]
