# Supplementary material for: Value of a BRAFV600E and lymphocyte subset-based nomogram for discriminating benign lesions from papillary thyroid carcinoma in C-TIRADS 3 and higher nodules
Source: Front Endocrinol (Lausanne). 2025 Aug 15;16:1608222. doi: 10.3389/fendo.2025.1608222 (PMC12394038; doi:10.3389/fendo.2025.1608222)
Supplement: Supplementary Table 1 — Assignment Table of variables. [file Table1.docx]

**Supplementary Table 1. Assignment Table of variables**

| variables | assignments |
| --- | --- |
| sex | 0:woman;1:man |
| BRAF^V600E^ genotype | 0:wild type;1:mutant type |
| size | 0:size<10mm;1:size≥10mm |
| echogenicity | 0:non-hypoechoic;1:hypoechoic |
| composition | 0:non-solid; 1:solid |
| multifocality | 0:solitary; 1:multiple |
| boundary | 0:clear; 1:obscure |
| Morphology | 0:regular; 1:irregular |
